# Abnormal flow pattern of low wall shear stress and high oscillatory shear index in spontaneous vertebral artery dissection with vertebral artery hypoplasia

**DOI:** 10.3389/fnins.2023.1179963

**Published:** 2023-06-14

**Authors:** Jiajia Bao, Xinling Gan, Wentao Feng, Yanbo Li, Yue Qiu, Muke Zhou, Jian Guo, Li He

**Affiliations:** ^1^Department of Neurology, West China Hospital, Sichuan University, Chengdu, China; ^2^Key Laboratory of Biomechanics and Mechanobiology (Beihang University) Ministry of Education, Beijing Advanced Innovation Center for Biomedical Engineering, School of Biological Science and Medical Engineering, Beihang University, Beijing, China; ^3^Department of Applied Mechanics, Sichuan University, Chengdu, China

**Keywords:** spontaneous vertebral artery dissection, vertebral artery hypoplasia, hemodynamic characteristics, blood flow patterns, computational fluid dynamics, nitric oxide production rate

## Abstract

**Introduction:**

Spontaneous vertebral artery dissection (sVAD) might tend to develop in vertebral artery hypoplasia (VAH) with hemodynamic dysfunction and it is crucial to assess hemodynamics in sVAD with VAH to investigate this hypothesis. This retrospective study aimed to quantify hemodynamic parameters in patients with sVAD with VAH.

**Methods:**

Patients who had suffered ischemic stroke due to an sVAD of VAH were enrolled in this retrospective study. The geometries of 14 patients (28 vessels) were reconstructed using Mimics and Geomagic Studio software from CT angiography (CTA). ANSYS ICEM and ANSYS FLUENT were utilized for mesh generation, set boundary conditions, solve governing equations, and perform numerical simulations. Slices were obtained at the upstream area, dissection or midstream area and downstream area of each VA. The blood flow patterns were visualized through instantaneous streamline and pressure at peak systole and late diastole. The hemodynamic parameters included pressure, velocity, time-averaged blood flow, time-averaged wall shear stress (TAWSS), oscillatory shear index (OSI), endothelial cell action potential (ECAP), relative residence time (RRT) and time-averaged nitric oxide production rate (TAR_NO_).

**Results:**

Significant focal increased velocity was present in the dissection area of steno-occlusive sVAD with VAH compared to other nondissected areas (0.910 m/s vs. 0.449 vs. 0.566, *p* < 0.001), while focal slow flow velocity was observed in the dissection area of aneurysmal dilatative sVAD with VAH according to velocity streamlines. Steno-occlusive sVAD with VAH arteries had a lower time-averaged blood flow (0.499 cm^3^/s vs. 2.268, *p* < 0.001), lower TAWSS (1.115 Pa vs. 2.437, *p =* 0.001), higher OSI (0.248 vs. 0.173, *p* = 0.006), higher ECAP (0.328 Pa^−1^ vs. 0.094, *p* = 0.002), higher RRT (3.519 Pa^−1^ vs. 1.044, *p* = 0.001) and deceased TAR_NO_ (104.014 nM/s vs. 158.195, *p* < 0.001) than the contralateral VAs.

**Conclusion:**

Steno-occlusive sVAD with VAH patients had abnormal blood flow patterns of focal increased velocity, low time-averaged blood flow, low TAWSS, high OSI, high ECAP, high RRT and decreased TAR_NO_. These results provide a good basis for further investigation of sVAD hemodynamics and support the applicability of the CFD method in testing the hemodynamic hypothesis of sVAD. More detailed hemodynamic conditions with different stages of sVAD are warranted in the future.

## Introduction

Spontaneous vertebral artery dissection (sVAD) occurs via a non-traumatic tearing of the arterial wall and is the second leading cause of stroke in young and middle-aged adults (Schievink, 2001; Ferro et al., 2010). However, the pathogenesis of sVAD remains obscure (Schievink, 2001; Debette and Leys, 2009). Accumulating evidence suggests that vertebral artery hypoplasia (VAH) is a significant independent risk factor for sVAD, as found in our previous study (Zhou et al., 2015; Günther et al., 2016). Asymmetric hemodynamics resulting from VAH make it more likely to cause ischemic events (Giannopoulos et al., 2007; Hong et al., 2009). Hence, we hypothesized that sVAD would tend to develop in VAH with hemodynamic dysfunction.

However, the complexity of the hemodynamic microenvironment in the vertebrobasilar system and the lack of *in vivo* models of sVAD and VAH that evaluate detailed hemodynamics have made it difficult to explicitly verify this hypothesis (Wake-Buck et al., 2012). Although previous studies used ultrasound and magnetic resonance imaging (MRI) to acquire hemodynamic data of sVAD and VAH, the accuracy of these measures is controversial (Augst et al., 2003; Giannopoulos et al., 2007; Hori et al., 2020). Therefore, it is crucial to realistically assess hemodynamic parameters in sVAD with VAH arteries to investigate this hypothesis. Computational fluid dynamics (CFD) has been widely utilized to analyze *in vivo* hemodynamics of healthy and diseased arteries because this technique allows the observation of flow patterns and quantification of hemodynamic parameters via simulation of a constructive model of patient-specific imaging data (Biasetti et al., 2010; Samady and Jaber, 2022).

To quantify hemodynamic parameters and investigate the potential hemodynamic characteristics of sVAD with VAH arteries, we performed CFD to reconstruct three-dimensional (3D) models and analyze the numerical simulation of sVAD with VAH arteries based on CT angiography (CTA) data. We further compared these hemodynamic parameters to contralateral healthy VAs, with the aim of providing direct and reliable evidence of hemodynamic characteristics in sVAD with VAH arteries and establishing a solid foundation for further investigations into sVAD hemodynamics.

## Methods

### Participants

Between October 1, 2014, and July 31, 2019, we retrospectively enrolled patients who suffered posterior circulation infarcts due to an sVAD of the hypoplastic VA in the Neurology department of West China Hospital. The Ethical Review Committee of the West China Hospital of Sichuan University approved this research [2020(69)]. Since this study is a retrospective study collecting de-identified data, the requirement of obtaining informed consent was waived. The following inclusion criteria were used for patient selection: (1) diagnosis of sVAD and VAH using previously described criteria (Maruyama et al., 2012; Zhou et al., 2015); (2) posterior circulation infarcts attributed to sVAD diagnosis based on clinical and radiological data (Adams et al., 1993); (3) sVAD on the side of hypoplastic VA; and (4) source images of CTA (DICOM format) before treatment. The following exclusion criteria were used: (1) the presence of multiple dissections in cervical arteries; (2) the presence of nondissected aneurysms or nondissected stenosis in cervical arteries; (3) history of associated connective tissue or vascular disorders; (4) history of head or neck trauma; (5) cervical intracranial artery malformation; (6) cervical and intracranial artery atherosclerosis; (7) time of sVAD onset to hospitalization was longer than 2 months (Schievink, 2001); and (8) incomplete information (e.g., poor 3D models because of insufficient quality of CTA imaging). We recorded detailed demographic and clinical characteristics, including lesion morphological subtypes. Stroke severity was assessed with the National Institutes of Health Stroke Scale (NIHSS) score on admission. Clinical outcome was evaluated with the modified Rankin Scale (mRS) at 3-month during clinical follow-up by face-to-face interview or telephone.

### Computational fluid dynamics

Thin-slice CTA images of the sVAD with VAH arteries were obtained, and 3D models before treatment were reconstructed based on these CTA images (DICOM format) using the commercially available software Mimics (version 20.0; Materialise, NV, Belgium) and Geomagic Studio software (Geomagic, Research Triangle Park, NC, United States). The final reconstructed models covered the aortic arch from the initial basilar artery (posterior circulation). The model had one inlet (aortic ostium) and six outlets (cross-sections of main arteries in the considered region), as suggested previously (Jozwik and Obidowski, 2010). The inlet and outlets were cut orthogonally to the centerline and extended 10 times the vessel diameter to ensure that the boundary condition would not influence the flow field within the vessels. As previously described, velocity in the inlet is 1.44 m/s and all static pressures in all outlet cross-sections were equal to 13 kPa. The portion of the arteries included in the geometrical model was determined by the quality of the images. The walls of all vessels were assumed to be rigid and nondeformable with changes in the pressure of the blood to simulate the flow.

Reconstructed models were imported as a stereolithography (STL) format file into ANSYS ICEM (version 19.0; Fluent, Inc., Lebanon, NH, United States) for mesh generation. A mesh sensitivity analysis that compared pressure, mass flow, and wall shear stress (WSS) at coarse, middle and fine mesh was generated to obtain a relationship between discretization error and element size and an estimate for the required element size (Biasetti et al., 2010). The mesh sensitivity analysis did not yield any significant differences that could influence the results of the computations performed (Supplementary Table S1). To account for time-consuming transient simulations, a mid-size density (2.72 × 10^6^ elements) was used. A mesh file was generated and exported.

Similar to a previous study (Jozwik and Obidowski, 2010), a constant density of blood equal to 1,055 kg/m^3^ was assumed. Blood is mathematically modeled as an incompressible, laminar and nonNewtonian fluid. The modified Power Law model was used to express blood viscosity. Detailed calculations of the inlet and outlet conditions were performed using the same previously described method, which resulted in a stable CFD simulation (Jozwik and Obidowski, 2010). Due to the nonstationary nature of blood flow, turbulence is expected in many places of the system being modeled. To meet the needs of the simulation, a shear stress transport (SST) model was used as the turbulence model.

All CFD simulations were performed in ANSYS FLUENT (version 19.0; Fluent, Inc., Lebanon, NH, United States) using standard numeric techniques. The governing equations of blood flow are the continuity and Navier–Stokes equations, which are described as:


(1) $$\nabla •\vec{v}=0$$

(2) $$\rho \left(\frac{\partial \vec{v}}{\partial \vec{t}}+\vec{v}.\nabla \vec{v}\right)=-\nabla p+\nabla .\left[\mu \left(\nabla \vec{v}+{\left(\nabla \vec{v}\right)}^{T}\right)\right]+f$$

where $\vec{v}$ and P represent the velocity and pressure vectors, respectively, and ρ (1,055 kg/m^3^) and μ (0.0035 Pa·s) denote the density and dynamic viscosity of blood, respectively.

Pressure-implicit splitting of operators (PISO) for the pressure–velocity coupling and a second-order upwind scheme for the momentum spatial discretization were used to calculate the hemodynamic parameters. Five cardiac cycles were calculated to ensure periodicity and achieve stable solutions, with a time step of 0.0025 s, and the data obtained for the fifth cardiac cycle were used in subsequent analyses.

Moreover, slices at the upstream normal area of sVAD, the area of sVAD, and the downstream normal area of sVAD for each patient were obtained. For healthy VAs, three slices (upstream area, midstream area, and downstream area) corresponding to the contralateral sVAD were also acquired (Supplementary Figure S1).

### Patterns of blood flow analysis and hemodynamic parameters

The patterns of blood flow included the instantaneous streamline and the pressure at peak systole (0.2 s) and late diastole (0.7 s). The maximum velocity and maximum pressure of different slices at peak systole were calculated. To better present a comparison of pulsatile and steady-state flows, the time-averaged blood flow for each VA was calculated as:

(3) $$\emptyset_{a}=\frac{1}{T}\overset{T}{\underset{0}{\int }}\emptyset dt$$

∅ is the scalar flow at each node of the computational grid.

We also analyzed the WSS-related indices. The average WSS of each vessel wall cell over a cardiac cycle was evaluated using the time-averaged wall shear stress (TAWSS), defined as:


(4) $$\mathrm{TAWSS=}\frac{1}{T}\overset{T}{\underset{0}{\int }}\mathrm{WSS}\left(\mathrm{s,t}\right)\;\;dt$$

where T is the cardiac cycle period, WSS is the instantaneous WSS vector, and s is the position on the vessel wall.

The cyclic departure of the WSS vector from its predominant axial alignment during the cardiac cycle is introduced by the oscillatory shear index (OSI), described as Ku et al. (1985):

(5) $$\mathrm{OSI=}0.5\left[1-\left(\frac{\left|\int_{0}^{T}WSS\left(s,t\right).dt\right|}{\int_{0}^{T}\left|WSS\left(s,t\right)\right|.dt}\right)\right]$$

where T is the cardiac cycle period, WSS is the instantaneous WSS vector, and s is the position on the vessel wall.

The degree of “thrombotic susceptibility” at the vessel wall is represented by the endothelial cell action potential (ECAP) and expressed as Kelsey et al. (2017):

(6) $$\mathrm{ECAP=}\frac{OSI}{TWASS}$$

The relative residence time (RRT) is a hemodynamic indicator of the luminal surface areas experiencing low and oscillating WSS and defined as Zhang et al. (2015):


(7) $$\mathrm{RRT=}\frac{1}{\left(1-2.OSI\right).TWASS}\;\;\frac{1}{\frac{1}{T}\left|\int_{0}^{T}WSS\left(s,t\right).dt\right|}$$

Time-averaged nitric oxide (NO) production rate (TAR_NO_) reflects the NO production rate of the endothelium and can be calculated as Andrews et al. (2010) and Li et al. (2019):

TAR_NO_=


(8) $$\frac{1}{t}\overset{t}{\underset{0}{\int }}R_{NO}\left(t\right)2.13+457.5\times \frac{1}{t}\overset{t}{\underset{0}{\int }}\left(\frac{\left|WSS\left(c,t\right)\right|}{\left|WSS\left(c,t\right)\right|+3.5}\right)\times dt$$

By considering a quantitative analysis, we evaluated the TAWSS, OSI, ECAP, RRT and TAR_NO_ using the area-averaged mean, as suggested previously (Zhang et al., 2015). All hemodynamic parameters were measured at the peak of the systolic phase. More details are provided in Supplementary material.

### Statistical analysis

All statistical analyses were performed using IBM SPSS Statistics software (version 22; IBM Corp, Armonk, NY, United States). To analyze the velocity and pressure of different slices (upstream area, dissection area or midstream area, downstream area), two-way analysis of variance (ANOVA) [least significant difference (LSD)] followed by Bonferroni’s *post-hoc* test and the Friedman test were used. Differences in hemodynamic parameters between the sVAD with VAH arteries and the contralateral healthy VAs were compared using the paired t-test or the Wilcoxon matched-pairs signed-rank test. Continuous variables are expressed as the means ± standard deviations (SDs) or medians (interquartile ranges), and categorical variables are described using the frequency and percent. For all statistical analyses, the significance level was set at *p* < 0.05.

## Results

### Study population

Per the inclusion and exclusion criteria, 14 patients with sVAD of the hypoplastic VA (12 males, 2 females) were included in this study. Their demographic and clinical characteristics are illustrated in Table 1. The median age of all patients was 36 years (with a range between 21 and 51 years). Of the 14 patients, 10 presented with right sVAD with VAH, while 4 presented with left sVAD with VAH. Steno-occlusive sVAD (12/14) was more frequently observed in the present study, and aneurysmal dilatative sVAD was found in only two patients (neither of whom had rupture). The most frequent segments of sVAD were the V3 and V4 levels, and the most common general clinical features was vertigo and dizziness. The ischemic brain regions attributed to sVAD included the dorsolateral medullary, dorsal medullary and cerebellar hemispheres.

**Table 1:** Demographic and clinical characteristics of patients who diagnosed with sVAD with VAH.

Patients (*n* = 14)	N (%)/median (IQR)
Gender	
Female	2 (14%)
Male	12 (86%)
Age (years)	36 (30–37)
Side of sVAD	
Right	10 (71%)
Left	4 (29%)
Segments of sVAD	
V1	1
V2	2
V3	5
V4	6
Subtypes of sVAD	
Steno-occlusion	12
Aneurysmal dilatation	2
Length of dissection (mm)	29.46 (19.09–32.78)
Clinical symptom	
Headache	6
Neck pain	2
Vertigo/dizziness	11
Dysarthria	3
Dysphagia	6
Nystagmus	2
Gait ataxia	6
Unilateral limb numbness	5
Unilateral limb paralysis	3
Brain infarct	
Dorsolateral medullary	10
Dorsal medullary	1
Cerebellar hemisphere	3
Onset to door time (day)	4 (3–7)
SBP at admission (mmHg)	131 (122–148)
DBP at admission (mmHg)	84 (79–92)
Risk factors	
Hypertension	1
Diabetes mellitus	2
Atrial fibrillation	0
Migraine	2
History of ischemic stroke	0
Hyperlipidemia	2
Current smoking	3
History of alcohol	2
Baseline NIHSS	2 (1–4)
mRS at 90 days	
0	8
≥1	6

sVAD, spontaneous vertebral artery dissection; VAH, Vertebral Artery Hypoplasia; SBP, systolic blood pressure; DBP, diastole blood pressure; NIHSS, National Institutes of Health Stroke Scale; mRS, modified Rankin Scale.

### Patterns of blood flow analysis

**Figure 1:**
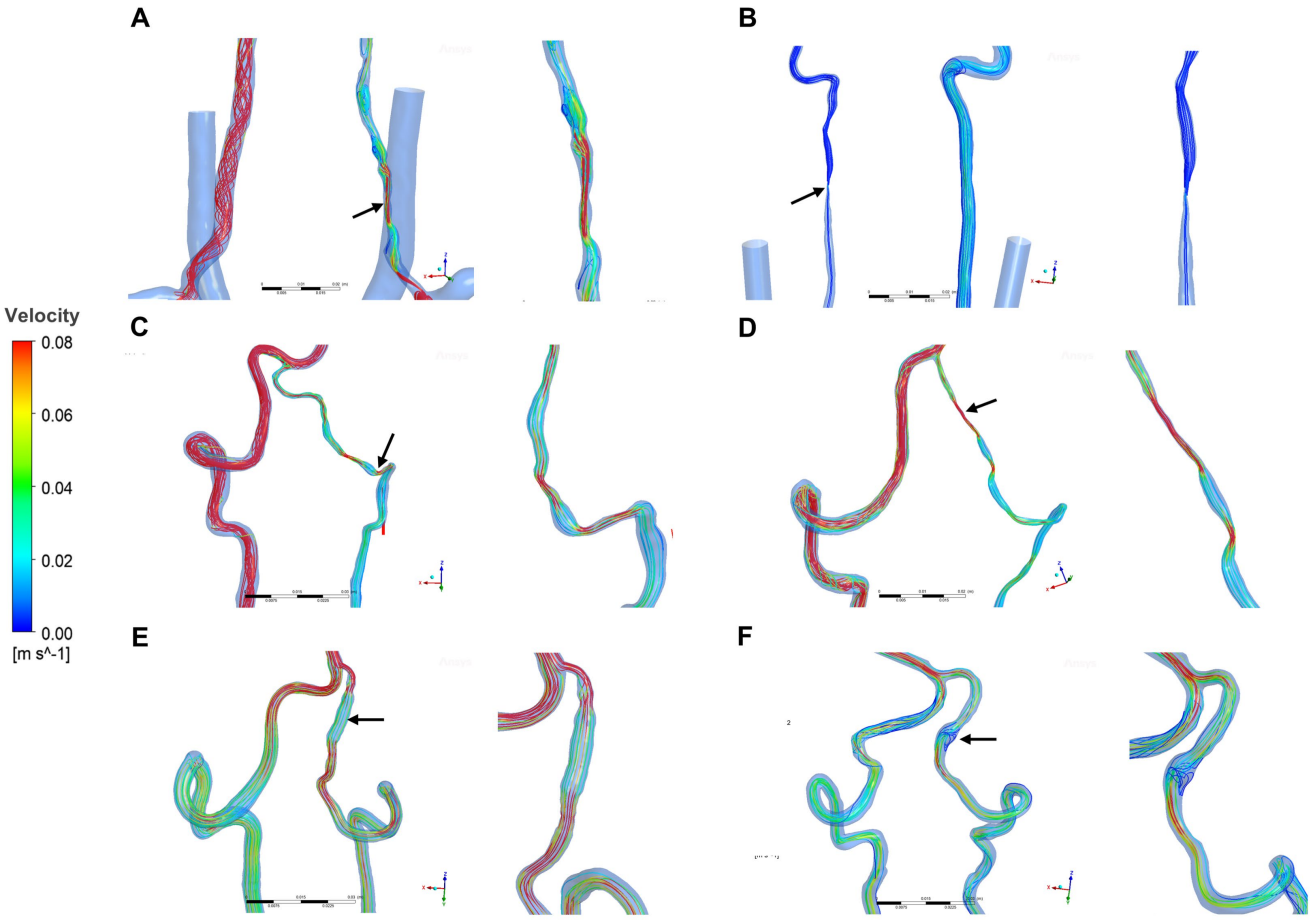
Velocity streamlines for sVAD with VAH arteries and contralateral normal VAs at peak systole. **(A–D)** Steno-occlusive sVAD with VAH arteries. **(E,F)** Aneurysmal dilatative sVAD with VAH artery. →: dissected region. sVAD, spontaneous vertebral artery dissection; VAH, vertebral artery hypoplasia; VAs, vertebral arteries.

Two hemodynamic variables (pressure and velocity) that directly observed blood flow patterns, were obtained from CFD simulations. Figure 1 demonstrated velocity streamlines at peak systole. We observed that laminar flow occurred in most healthy VAs at peak systole. More disordered flow was observed in the dissection area in sVAD with VAH arteries (Figure 1). We also observed that normal slices of steno-occlusive sVAD with VAH arteries had slower blood flow velocity than the opposite slices of contralateral healthy (Figures 1A–D). Aneurysmal dilatative sVAD cases manifested slow blood flow velocity in the dissection region, but the velocity was not visibly different between sVAD with VAH artery and contralateral normal VA in those cases (Figures 1E,F). The mean maximum velocity values for the VAs with the upstream area, dissection/midstream area, and downstream area are listed in Table 2. Friedman test for multiple comparisons revealed significant differences in the maximum velocity of steno-occlusive sVAD with VAH arteries (χ^2^ = 19.500, *p* < 0.001) among the upstream normal area (0.449 m/s ± 0.456), dissection area (0.910 m/s ± 0.738), and downstream normal area (0.566 m/s ± 0.646), whereas maximum velocity between the 3 slices of healthy VAs was not statistically significant (*F* = 0.711, *p* = 0.502) (Table 2).

**Table 2:** The upstream area, dissection area or midstream area, and downstream area of hemodynamic parameters in sVAD with VAH and contralateral normal VAs.

Slices	Steno-occlusive sVAD with VAH			Healthy VAs		
	Velocity (m/s)^‡^	Pressure (Pa)^‡^	WSS(Pa)^‡^	Velocity (m/s)^#^	Pressure (Pa)^‡^	WSS(Pa)^‡^
Upstream	0.449 ± 0.456	15273.458 ± 2194.785	4.100 ± 7.148	0.727 ± 0.469	15474.868 ± 2332.914	6.422 ± 5.828
Dissection area/Midstream	0.910 ± 0.738	14913.067 ± 2117.591	11.667 ± 14.393	0.881 ± 0.578	15252.871 ± 2244.378	10.739 ± 8.699
Downstream	0.566 ± 0.646	14504.092 ± 1844.347	7.397 ± 9.186	0.825 ± 0.642	14859.277 ± 2101.296	12.021 ± 14.912
*p* value	<0.001***	<0.001***	<0.01**	0.502	0.017*	0.472

*p < 0.05; **p < 0.01; ***p < 0.001. ^#^Two-way analysis of variance (ANOVA). ^‡^Friedman test.

Variables were expressed as $\bar{x}$ ± s.

VA, vertebral arteries; sVAD, spontaneous vertebral artery dissection; WSS, wall shear stress.

**Figure 2:**
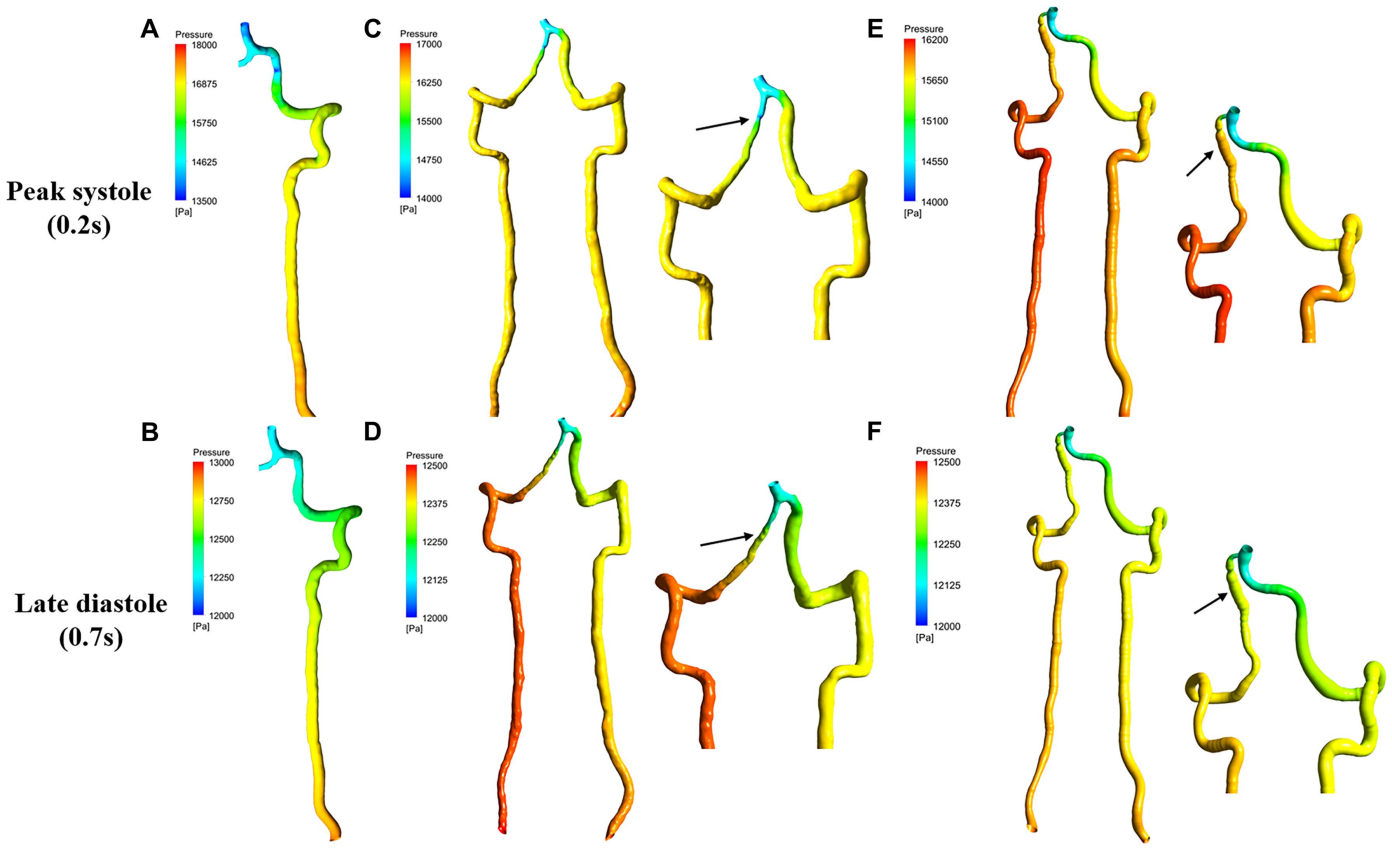
Pressure contour map for sVAD with VAH arteries and contralateral normal VAs at peak systole and late diastole. **(A,B)** Contralateral healthy VAs. **(C,D)** Steno-occlusive sVAD with VAH arteries. **(E,F)** Aneurysmal dilatative sVAD with VAH artery. →: dissected region. sVAD, spontaneous vertebral artery dissection; VAH, vertebral artery hypoplasia; VAs, vertebral arteries.

Figure 2 presents the pressure distribution superimposed with pressure contour map at peak systole and late diastole. As shown in Figure 2, pressure was not visibly different between steno-occlusive dissection region of sVAD and VAH artery and the midstream of the contralateral normal VA. Regarding the aneurysmal dilatation type, focal high pressure was observed in the aneurysmal dissection region of sVAD with VAH artery according to pressure contour map (Figure 2), but we did not perform statistical analyses due to the limited sample size. Moreover, whether for healthy VAs or sVAD with VAH arteries, the pressure from inlet to outlet showed a gradually dropping trend in healthy VAs and sVAD with VAH arteries. Friedman test also showed that the pressure dropping trend in the healthy VAs and steno-occlusive sVAD with VAH arteries were statistically significant (χ^2^ = 8.167, *p* = 0.017 and χ^2^ = 17.167, *p* < 0.001, respectively) (Table 2).

### Hemodynamics characteristics

**Figure 3:**
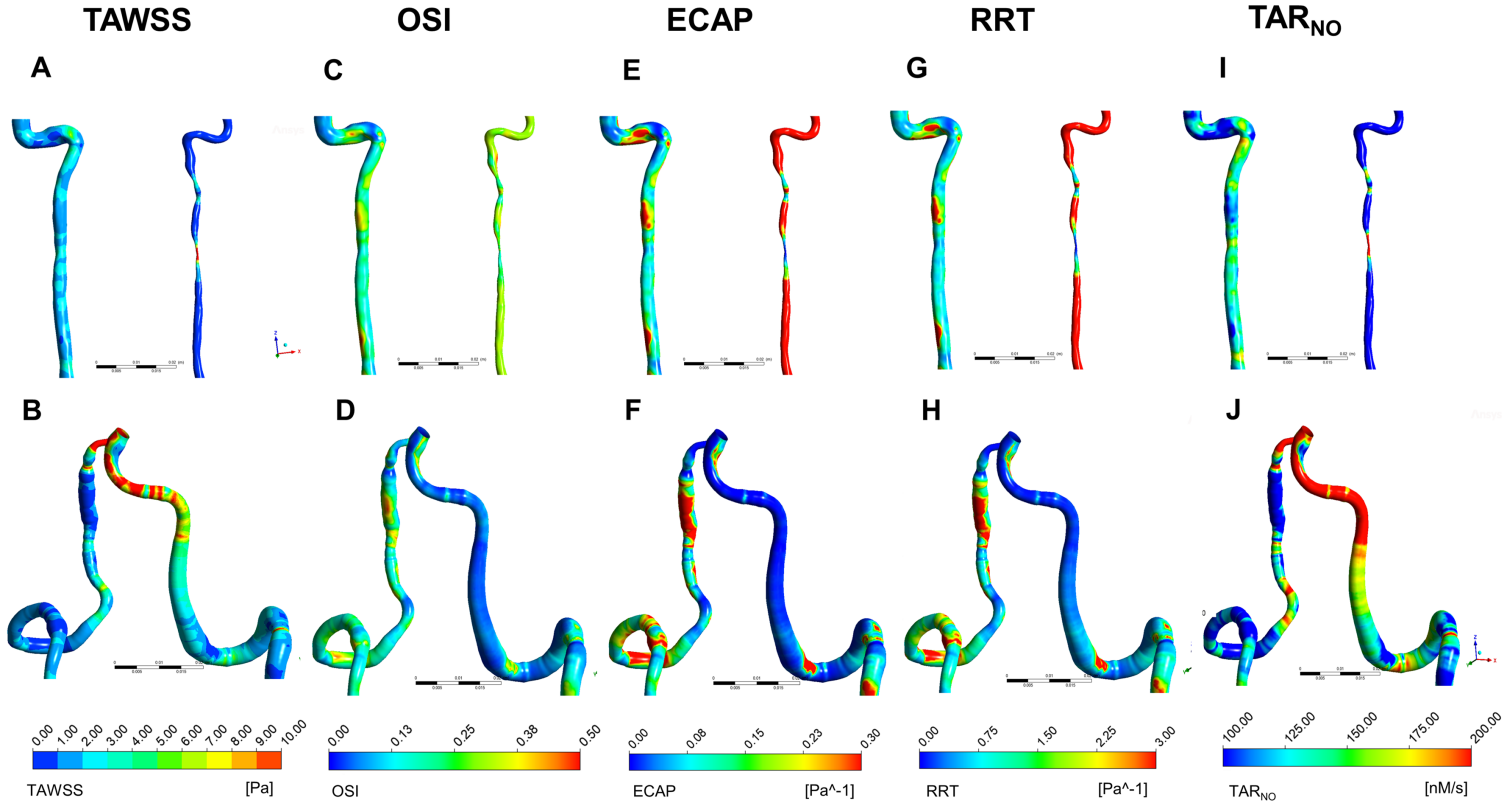
Distribution of TWASS, OSI, ECAP, RRT, and TAR_NO_ at peak systole for sVAD with VAH arteries and contralateral normal VAs. **(A,B)** Distribution of TWASS; **(C,D)** distribution of OSI; **(E,F)** distribution of ECAP; **(G,H)** distribution of RRT; **(I,J)** distribution of TAR_NO_. **(A,C,E,G,I)** Steno-occlusive sVAD with VAH; **(B,D,F,H,J)** aneurysmal dilatative sVAD with VAH. →: dissected region. TWASS, time-averaged wall shear stress; OSI, oscillatory shear index; ECAP, endothelial cell action potential; RRT, relative residence time; TAR_NO_, time-averaged nitric oxide production rate; sVAD, spontaneous vertebral artery dissection; VAH, vertebral artery hypoplasia; VAs, vertebral arteries.

To assess the hemodynamic characteristics of sVAD with VAH arteries, time-averaged blood flow, TWASS, OSI, ECAP, RRT, and TAR_NO_ of each VA were solved. Figure 3 presents the distribution of TAWSS, OSI, ECAP, RRT, and TAR_NO_ on the vessel wall. TAWSS contour plots are shown in Figures 3A,B. Steno-occlusive sVAD patients exhibited local maxima of TAWSS in the dissection area (Figure 3A). For the two patients with aneurysmal dilatative sVAD, the dissection area manifested low TAWSS (Figure 3B). Other non-dissection areas of sVAD with VAH arteries had lower TAWSS than the counterparts of the contralateral healthy VAs regardless of the dissection subtype. The OSI contour plots (Figures 3C,D) showed that the local maxima of OSI were distributed in the dissection area in the steno-occlusive VAD model (Figure 3C) and aneurysmal dilatative VAD model (Figure 3D). Moreover, there was higher OSI on the sVAD with VAH artery regardless of dissection subtypes in comparison with contralateral healthy VA. Most of the walls of the sVAD with VAH artery showed a relatively high ECAP and high RRT (Figures 3E–H), and the distributions of high ECAP and high RRT were approximately same. As Figure 3I shown, the highest TAR_NO_ concentration was observed at dissection area in steno-occlusive subtype. In contrast, the local minimum TAR_NO_ concentration occurred at the dissection area in aneurysmal dilatation subtype (Figure 3J). In non-dissection area, both of steno-occlusive sVAD and aneurysm dilatative sVAD had lower TAR_NO_ concentration compared with the opposite healthy VAs.

The quantitative results of time-averaged blood flow, TAWSS, OSI, ECAP, RRT and TAR_NO_ are recorded in Table 3. All steno-occlusive sVAD cases showed strong statistically significant differences between all hemodynamic parameters between the sVAD artery and non-sVAD artery. Compared with contralateral healthy VAs, steno-occlusive sVAD with VAH arteries had a lower time-averaged blood flow (0.499 cm^3^/s ± 0.467 vs. 2.268 ± 0.984, *t* = −7.381, *p* < 0.001), lower TAWSS (1.115 Pa ± 0.589 vs. 2.437 ± 0.970, *t* = −4.287, *p* = 0.001), higher OSI (0.248 ± 0.054 vs. 0.173 ± 0.038, *t* = 3.382, *p* = 0.006), higher ECAP (0.328 Pa^−1^ ± 0.192 vs. 0.094 ± 0.034, *t* = 4.143, *p* = 0.002), higher RRT (3.519 Pa^−1^ ± 1.960 vs. 1.044 ± 0.407, *t* = 4.216, *p* = 0.001) and deceased TAR_NO_ (104.014 nM/s ± 28.308 vs. 158.195 ± 18.082, *t* = −6.021, *p* < 0.001) according to statistical analyses. More details are provided in Supplementary material.

**Table 3:** Comparisons of hemodynamic parameters between steno-occlusive sVAD with VAH and contralateral normal VAs.

Parameters	Steno-occlusive sVAD with VAH	Normal VAs	*t*	*p-*value
TWASS(Pa)	1.115 ± 0.589	2.437 ± 0.970	−4.287	0.001**
OSI^†^	0.248 ± 0.054	0.173 ± 0.038	3.382	0.006**
ECAP(Pa^−1^)	0.328 ± 0.192	0.094 ± 0.034	4.143	0.002**
RRT(Pa^−1^)	3.519 ± 1.960	1.044 ± 0.407	4.216	0.001**
TAR_NO_ (nM/s)	104.014 ± 28.308	158.195 ± 18.082	−6.021	<0.001***
Time-averaged blood flow (cm^3^/s)	0.499 ± 0.467	2.268 ± 0.984	−7.381	<0.001***
Cross-sectional area (cm^2^)	5.514 ± 2.465	13.294 ± 3.355	−14.185	<0.001***

**p < 0.01; ***p < 0.001. Variables were expressed as $\bar{x}$ ± s.

VA, vertebral arteries; sVAD, spontaneous vertebral artery dissection; VAH, vertebral artery hypoplasia; TWASS, time-averaged wall shear stress; OSI, oscillatory shear index; ECAP, endothelial cell action potential; RRT, Relative residence time; TAR_NO_, time-averaged NO production rate.

## Discussion

The present study used CFD to reconstruct 28 high-quality 3D models of VA, display blood flow patterns, and quantify hemodynamic parameters of sVAD with VAH arteries, which allowed us to detect the occurrence of abnormal blood flow patterns, smaller time-averaged blood flow, lower TAWSS, higher OSI, higher ECAP, higher RRT and decreased TAR_NO_ in steno-occlusive sVAD with VAH arteries. These results provided direct hemodynamic characteristics in sVAD with VAH arteries and supported the applicability of the CFD method in testing the hemodynamic hypothesis of sVAD. In contrast to prior studies (Giannopoulos et al., 2007; McNally et al., 2018), this study is one of the first biomechanical studies of the hemodynamic pathogenesis of sVAD to apply the CFD method.

First, we quantified blood flow velocity and pressure, that was different from other prior VAD hemodynamics studies (de Bray et al., 1997; Zhang et al., 2021). We detected that a focal increase in blood flow velocity at the dissected area compared to other nondissected areas in steno-occlusive sVAD with VAH arteries, which is consistent with a previous ultrasound study of stenosis VAD (de Bray et al., 1997). In contrast, aneurysmal dilatative sVAD with VAH arteries showed a decreased blood flow velocity at the dissection area based on the velocity streamlines rather than statistical analyses. Additionally, there was more pronounced disordered flow in sVAD with VAH arteries regardless of the dissection subtype. We also observed a slower blood flow velocity in nondissected areas of steno-occlusive sVAD with VAH arteries than in the counterparts of contralateral healthy VAs. This phenomenon may have occurred because hypoplastic VAs have a lower mean flow velocity, which was previously found using Transcranial Doppler (TCD) (Min and Lee, 2007) and sVAD on the same side as hypoplastic VAs in the present study. We also found that dissected stenosis areas manifested low pressure due to a sudden narrowing of the inner diameter, and dissected aneurysmal dilation manifested high pressure based on the pressure contour map. However, there were no visibly differences in pressures between the dissected area of steno-occlusive sVAD with VAH arteries and the counterparts of contralateral healthy VAs. The small differences in pressure between the two groups were expected due to the limited sample size. Consequently, these pressure results of sVAD with VAH need further confirmation in a larger sample of patients to enable inter-patients comparisons and statistical analyses follow-up studies. Overall, our findings suggest that high velocity and focal low pressure occurred at the dissected stenosis region, while low velocity and focal high pressure occurred at the dissected aneurysm region. These results reveal the phenomena that different subtypes of sVAD possibly exhibited different blood flow patterns. Moreover, these observations also hint at an unstable hemodynamic microenvironment in sVAD with VAH arteries, and the unstable blood flow patterns may be a risk factor for vascular diseases (Biasetti et al., 2010; Sui et al., 2015). Hence, future investigations of sVAD hemodynamics should pay more attention to patient-specific blood flow patterns with morphologic and regional variation.

Furthermore, we detected a notably lower time-averaged blood flow in steno-occlusive sVAD with VAH arteries compared with the contralateral healthy VAs. This phenomenon represents the insufficient blood supply in steno-occlusive sVAD with VAH arteries, which may be due to the following reasons. Firstly, the definition of congenital VAH indicates that the diameter of sVAD with VAH arteries was smaller than that of the contralateral healthy VAs (Zhou et al., 2015). Secondly, a decreased blood flow velocity at the nondissected area in steno-occlusive sVAD with VAH arteries was observed. Poiseuille’s fourth-power radius law indicates that liquid flow through a vessel is proportional to the fourth power of the radius (Wilkin, 1989), which resulted in a lower blood flow was detected in these subjects. Importantly, several studies demonstrated a correlation between blood flow and artery remodeling. For example, Langille and O'Donnell (1986) and Lee and Langille (1991) measured the blood flow through the vessel lumen of rabbits and suggested that long-term, flow-induced reductions in arterial diameter were due to a structural modification of the artery wall rather than just sustained contractive smooth muscle. Based on these previous findings, we speculate that congenital VAH and long-term blood flow reductions have a potentially positive feedback relationship, wherein long-term low blood flow may promote VA remodeling. If the inner diameter of hypoplastic VAs decreases further, it may affect blood flow, and if these regulative changes reach an unbalanced state, it may induce hemodynamic dysfunction of VAH triggered by other hemodynamic forces or nonhemodynamic risk factors. Although our speculation of the potential hemodynamic dysfunction mechanism of VAH needs further in-depth research to corroborate, it may explain the prior observation that VAH is prone to the development of cerebrovascular events.

Especially, our study using CFD analysis has offered new perspectives on the precise evaluation of WSS-related indices in sVAD with VAH arteries, which is an improvement compared to the conventional hemodynamic assessment methods used in prior studies (Augst et al., 2003; Giannopoulos et al., 2007; Hori et al., 2020). Below is an elaborated summary of our findings. Initially, our finding indicated that TAWSS decreased in steno-occlusive sVAD with VAH arteries. The reduction in blood flow within these steno-occlusive sVAD with VAH arteries is likely responsible for this finding. WSS is considered as a crucial hemodynamic parameter for predicting the risk of arterial dissection (Li et al., 2019; Xu et al., 2020), and its correlation with blood flow has been investigated intensively since 1991. Langille and O'Donnell (1986) were the first to reported that vascular remodeling of common carotid arteries in a rabbit external carotid ligation model reduced the diameter due to decreased blood flow, and they suggested that the adaptation of flow-induced remodeling restored WSS to control levels. Two cell-based studies substantially showed that the maintenance of WSS at normal physiological levels appeared to involve the development of intima media thickening (IMT) and artery remodeling, resulting in alterations in blood flow (Korshunov and Berk, 2003; Korshunov et al., 2006). More importantly, abnormal WSS has a negative effect on endothelial function because low WSS leads to a proinflammatory, procoagulant surface via activation of proinflammatory and procoagulant transcription factors (Boon and Horrevoets, 2009; Meng et al., 2014). Therefore, flow-induced WSS plays an important role in artery remodeling and endothelial function, both of which are related to vascular diseases (Cibis et al., 2014; Frösen et al., 2019). We hypothesized that prolonged low TAWSS may also be a key trigger in the hemodynamic pathogenesis hypothesis of sVAD. Although this hypothesis requires stronger evidence to support in the future, our study tentatively verified that steno-occlusive sVAD with VAH arteries had low TAWSS. Further hemodynamic studies of sVAD should pursue more detailed data about WSS at different stages and types of arterial dissection to explore risk predictors of sVAD.

We also observed a significant increase in OSI, ECAP and RRT in the dissection area regardless of sVAD subtypes. And the difference in OSI, ECAP and RRT values between steno-occlusive sVAD with VAH arteries and contralateral healthy VAs has showed significance after statistical analysis. One possible reason for our findings was that sVAD with VAH artery had more disturbed flow conditions than contralateral healthy VAs. Previous literatures have reported that high OSI areas were commonly detected around vortex flow, which could induce more reactive oxygen species (ROS) production from endothelial cells more than laminar shear (Hwang et al., 2003; Hohri et al., 2021), thereby contribute to vascular diseases, such as artery deformation and atherosclerosis (Xu et al., 2020; Chen et al., 2022). Additionally, our results revealed that sVAD with VAH arteries might have higher thrombotic susceptibility at the hemodynamic level owing to both of high ECAP and high RRT were indicators for the risk of thrombogenesis as previously described (Zhang et al., 2015; Ong et al., 2019), which supporting antithrombosis treatment as the main therapeutic intervention in patients with sVAD in clinical practice (Sugiyama et al., 2013; CADISS Trial Investigators et al., 2015). However, whether high ECAP and high RRT help create an unfavorable hemodynamic environment and somehow promote sVAD progression, or whether this unfavorable hemodynamic environment results from sVAD are still unknown.

In addition, decreased TAR_NO_ on sVAD with VAH arteries was detected in the present study. This finding is consistent with previous studies on atherosclerosis stenosis, suggesting that stenotic VAD might have potential effects on inhibiting the NO production of endothelium (Liu et al., 2012). TAR_NO_ has been seen as an indicator of NO transport of endothelium in previous publications (Li et al., 2019). NO, as a key endothelium-divided substance, has diverse functions such as leucocyte adhesion, endothelial regeneration, and vascular relaxation (Busse et al., 1995; de Caterina et al., 1995; Napoli et al., 2006). Converging evidence has demonstrated that NO produced from endothelium is a signal in regulating vascular wall function and low NO concentration may contribute to restenosis and thrombosis (Knowles and Moncada, 1992; Busse et al., 1995; Liu et al., 2015). The finding of TAR_NO_ in the present study could offer valuable insight into sVAD hemodynamic pathogenesis, and whether such phenomena truly exist *in vivo* remains to be demonstrated in future research.

In brief, the strength of the current study is its demonstration of the potential of CFD as a valuable tool for investigating hemodynamic parameters in sVAD. Our study provides novel insights into the hemodynamics of sVAD with VAH arteries. In contrast to conventional hemodynamic assessment tools, CFD offers several advantages, including non-invasiveness, patient-specificity, high accuracy, and cost-effectiveness (Pontone and Rabbat, 2017). Numerous previous studies have utilized CFD to flexibly analyze the hemodynamics and construct personalized mathematical models of vascular diseases (Deyranlou et al., 2020; Jiang et al., 2022). In particularly, CFD could provide more precise and detailed information on flow patterns, such as WSS, which is often challenging to obtain through other imaging techniques. Moreover, with the advancement of artificial intelligence, CFD holds promise as a clinical tool for the future (Pontone and Rabbat, 2017; Randles et al., 2017).

There are some limitations that must be acknowledged in this study. First, the sample size was relatively small. This was due in part to the low prevalence of sVAD with VAH (Schievink, 2001), and also because the quantity of reconstructed high-quality 3D models was susceptible to the patients’ images quality. Therefore, we ultimately obtained 28 high-quality 3D models of VA. Inherent selection bias was inevitable. Second, while the focus on hemodynamic characteristics of sVAD with VAH arteries is a strength of this study, interpretation of hemodynamic mechanism of sVAD attributed to VAH is limited by lack of data on images prior to sVAD occurs. Interestingly, some recent CFD studies used the variance and mean curvature calculation method to successfully recover the parent artery back to its pre-aneurysm state (Le et al., 2013). If this recovery method is utilized in future study, the hemodynamic hypothesis of sVAD attributed to VAH could be certified. Third, owing to only 2 cases exhibited aneurysmal dilatation in our study, the hemodynamic characteristics of aneurysmal sVAD were observed by contour plots rather than stratified analysis in order to assure the sufficient statistical power. The generalizability of these observations in aneurysmal dilatative sVAD with VAH arteries requires further confirmation.

Despite these limitations, this study suggest that abnormal flow patterns are present in the dissection area of sVAD with VAH arteries, and these hemodynamic dysfunctions might trigger the arterial wall to develop dissection. As far as we know, this study is a prime example for the application of CFD for quantification of hemodynamic characteristics in sVAD. These results may have implications for the importance of considering VAH in the management of sVAD, as well as for the understanding of its pathogenesis. Further studies are needed to confirm these findings and explore the possibility of hemodynamic characteristics as a diagnostic and prognostic measurement for sVAD with VAH arteries. In our view, the simplification of the relationship between the hemodynamic variables and sVAD occurrence may provide a convenient method to predict the risk of VAH developing sVAD, and more aggressive hemodynamic monitoring should be utilized in VAH patients if the sVAD hemodynamic pathogenesis hypothesis is confirmed in future studies.

## Conclusion

In conclusion, this study has novelty at the methodological level and provides direct evidence of hemodynamic characteristics in sVAD with VAH arteries, thereby enhancing our understanding of the hemodynamic pathogenesis of sVAD attributed to VAH. However, further validation is needed to confirm the results, and more detailed hemodynamic conditions with different stages of sVAD are warranted in the future. Furthermore, noninvasive hemodynamic assessment might have the potential to serve as a monitoring tool for predicting sVAD risk and assist clinicians in formulating intervention strategies for VAH patients.

## Data availability statement

The raw data supporting the conclusions of this article will be made available by the authors, without undue reservation.

## Ethics statement

The studies involving human participants were reviewed and approved by the West China Hospital of Sichuan University Biomedical Research Ethics Committee. Written informed consent for participation was not required for this study in accordance with the national legislation and the institutional requirements.

## Author contributions

JB: data collection, analysis and interpretation of data, and manuscript writing. XG: data collection and data analysis. WF: data analysis and data interpretation. YL: critical revision of the manuscript. YQ: data analysis. MZ: study design. JG and LH: study design, critical revision of the manuscript for important intellectual content, and study supervision. All authors contributed to manuscript revision, read, and approved the submitted version.

## Funding

This work was supported by the Natural Science Foundation of China (Grant no. 81971162), Institute of Brain Science and Brain-Inspired Technology of West China Hospital, Sichuan University, China Postdoctoral Science Foundation (Grant no. 2020M673248 and 2021M692294), and Sichuan Science and Technology Program (Grant no. 2021YJ0437).

## Conflict of interest

The authors declare that the research was conducted in the absence of any commercial or financial relationships that could be construed as a potential conflict of interest.

## Publisher’s note

All claims expressed in this article are solely those of the authors and do not necessarily represent those of their affiliated organizations, or those of the publisher, the editors and the reviewers. Any product that may be evaluated in this article, or claim that may be made by its manufacturer, is not guaranteed or endorsed by the publisher.

## Abbreviations

sVAD: spontaneous vertebral artery dissection
VAH: vertebral artery hypoplasia
VAs: vertebral arteries
MRI: magnetic resonance imaging
CFD: computational fluid dynamics
3D: three-dimensional
CTA: CT angiography
NIHSS: the National Institutes of Health Stroke Scale
mRS: the modified Rankin Scale
STL: stereolithography
WSS: wall shear stress
SST: shear stress transport
PISO: pressure-implicit splitting of operators
TAWSS: time-averaged wall shear stress
OSI: oscillatory shear index
ECAP: endothelial cell action potential
RRT: relative residence time
NO: nitric oxide
TAR_NO_: time-averaged nitric oxide production rate
ANOVA: analysis of variance
LSD: least significant difference
SDs: standard deviations
IMT: intima media thickening
ROS: reactive oxygen species
