# Supplementary material for: Abnormal flow pattern of low wall shear stress and high oscillatory shear index in spontaneous vertebral artery dissection with vertebral artery hypoplasia
Source: Front Neurosci. 2023 Jun 14;17:1179963. doi: 10.3389/fnins.2023.1179963 (PMC10303804; doi:10.3389/fnins.2023.1179963)
Supplement: Supplementary file 1 [file Data_Sheet_1.docx]

**Table S1.** Mesh sensitivity analysis

| Element size | Cells (×10^6^ ) | $\boldsymbol{\Delta}P$ error (%) | Mass flow error (%) | WSS error (%) | Left WSS error (%) |
| --- | --- | --- | --- | --- | --- |
| Coarse | 1.48 | 16.42 | 16.70 | 14.61 | 28.06 |
| Middle | 2.72 | 2.07 | 2.82 | 5.94 | 6.78 |
| Fine | 4.06 | - | - | - | - |

WSS = wall shear stress.


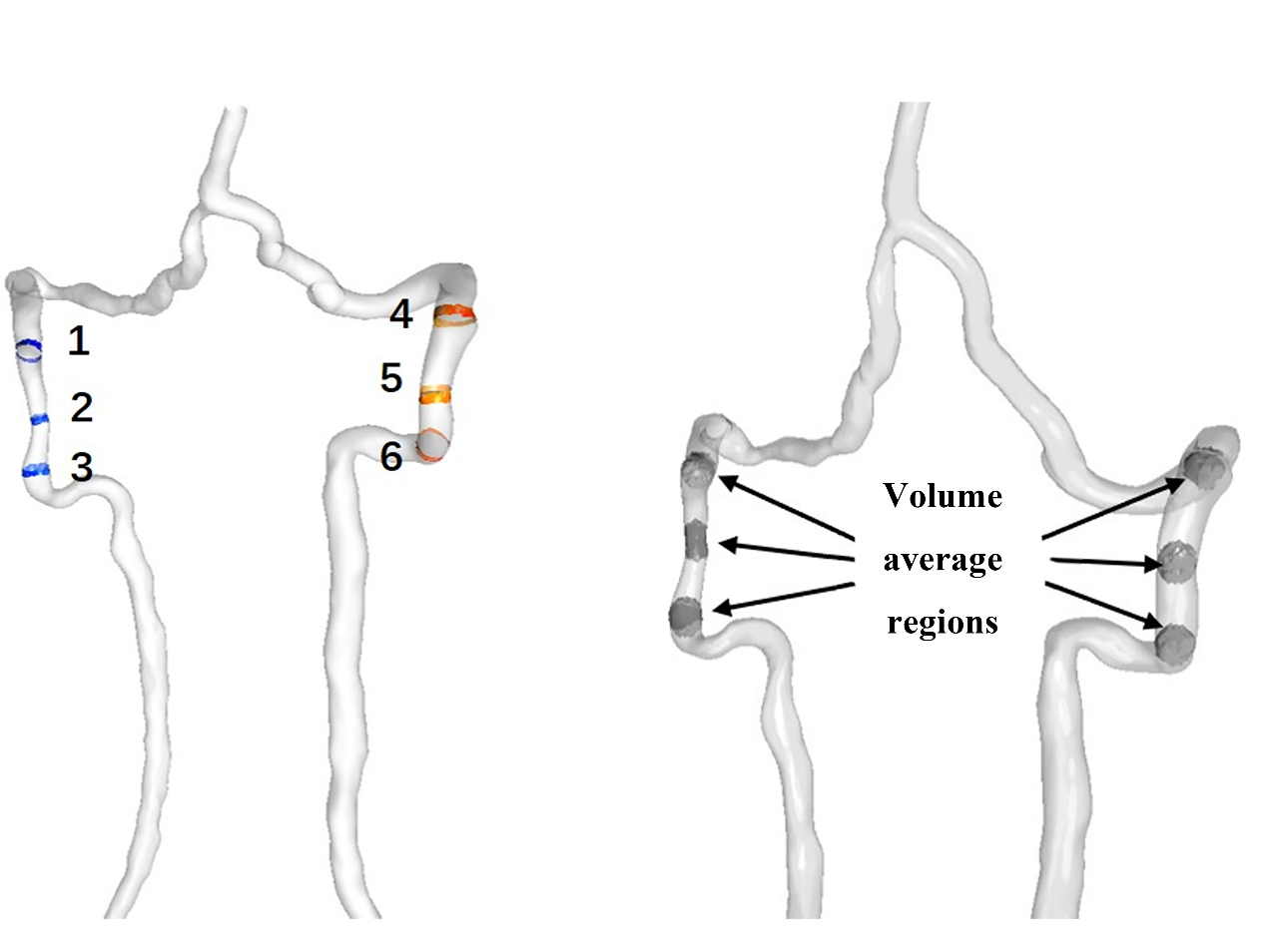


**Figure S1.** Slices at the upstream area, the area of sVAD or midstream area of healthy VAs, and the downstream area.

sVAD = spontaneous vertebral artery dissection; VAs = vertebral arteries.
